# Mitigating adverse outcomes in tuberculosis treatment: analyzing a non-compliance risk assessment strategy in a case report

**DOI:** 10.1590/S1678-9946202466059

**Published:** 2024-10-11

**Authors:** Carolina Rossoni de Melo, Cláudia Elizabeth Volpe-Chaves, Kássia Raquel da Silva, João Gabriel Cibolli Roso, Alexandre Albuquerque Bertucci, Eunice Atsuko Totumi Cunha, James Venturini, Ursulla Vilella Andrade, Michelle Mocellin Peruzzo, Wanessa da Silva Peres Bezerra, Sandra Maria do Valle Leone de Oliveira, Anamaria Mello Miranda Paniago

**Affiliations:** 1 Hospital Universitário Maria Aparecida Pedrossian, Programa de Residência Médica em Doenças Infecciosas e Parasitárias, Campo Grande, Mato Grosso do Sul, Brazil; 2 Hospital Universitário Maria Aparecida Pedrossian, Campo Grande, Mato Grosso do Sul, Brazil; 3 Hospital Regional de Mato Grosso do Sul, Campo Grande, Mato Grosso do Sul, Brazil; 4 Universidade Federal de Mato Grosso do Sul, Campo Grande, Mato Grosso do Sul, Brazil; 5 Universidade Federal de Mato Grosso do Sul, Programa de Pós-Graduação em Doenças Infecciosas e Parasitárias, Campo Grande, Mato Grosso do Sul, Brazil; 6 Laboratório Central de Mato Grosso do Sul, Campo Grande, Mato Grosso do Sul, Brazil; 7 Fundação Oswaldo Cruz, Campo Grande, Mato Grosso do Sul, Brazil

**Keywords:** Pulmonary tuberculosis, Treatment adherence, Mycobacterium tuberculosis, Risk factors

## Abstract

Tuberculosis (TB) is a global public health concern and a leading cause of death. Its persistence occurs mainly because barriers in the care cascade are not being fully addressed. Healthcare professionals and scientists have been addressing treatment challenges such as abandonment and irregular drug intake via strategies such as directly observing treatment and singular therapeutic projects to improve adherence. However, while protocols and guidelines advocate these strategies, their implementation requires a broader approach from healthcare teams. This article examines the importance of such strategies in clinical TB management and analyzes an unfavorable outcome in an immunocompetent patient treated for pulmonary tuberculosis (PTB) from 2017 to 2022. After recurrence and treatment, the patient continued to have persistent acid-fast bacilli in the sputum, positive cultures for *Mycobacterium tuberculosis*, and progressive lung lesions, despite receiving the recommended treatment. Although categorized as having an intermediate risk of treatment abandonment, the patient faced challenges, such as the COVID-19 pandemic, pregnancy, and being diagnosed with COVID-19. After therapeutic failure and the loss of beneficial prospects, palliative care was initiated. This case illustrates the complexities of managing TB in patients with recurrent disease despite apparent adherence to treatment. After reassessing the risk of abandonment score, the patient was categorized as high-risk. This underscores the importance of singular therapeutic projects, such as psychological support for high-risk or intermediate patients, to prevent negative outcomes. This case reinforces the critical need for comprehensive patient-centered approaches to successfully treat and manage TB.

## INTRODUCTION

Although treatable with medication that is free in several parts of the world, including Brazil, tuberculosis (TB) is still a major cause of mortality in healthcare services, even after the implementation of a cascade of care that is often not overcome^1^. The challenges of treatment abandonment and irregular drug treatment have motivated professionals and scientists to search for solutions, such as directly observed treatment (DOT) and singular therapeutic projects (STP)^2^.

Treatment dropout scores can be used to identify patients who require special attention to strengthen bonds and promote treatment adherence^3^. Our center has explored the use of this tool, which employs adapted scores for sex, age, drug use, smoking, religion, occupation, and previous TB treatment, to prioritize patients at higher risk of abandonment. Each variable is scored from 0 to 25, with a maximum of 100 points. A score below 20 is considered low risk, a score from 20 to 40 is intermediate risk, and a score of 45 or above is high risk^4^. However, despite being established in protocols and guidelines^5^, the implementation of this decision-making tool requires a more comprehensive approach by healthcare teams.

In this study, we explore the importance of strategies to ensure TB treatment adherence, based on a case report and an assessment of the risk of treatment abandonment in clinical management. We also address the challenges associated with unfavorable outcomes in a highly treatable disease.

### Ethics

Approval was obtained from the Research Ethics Committee of the Federal University of Mato Grosso do Sul (Nº 1.840.731). Patient signed an informed consent form before participating.

## CASE REPORT

**Figure 1:**
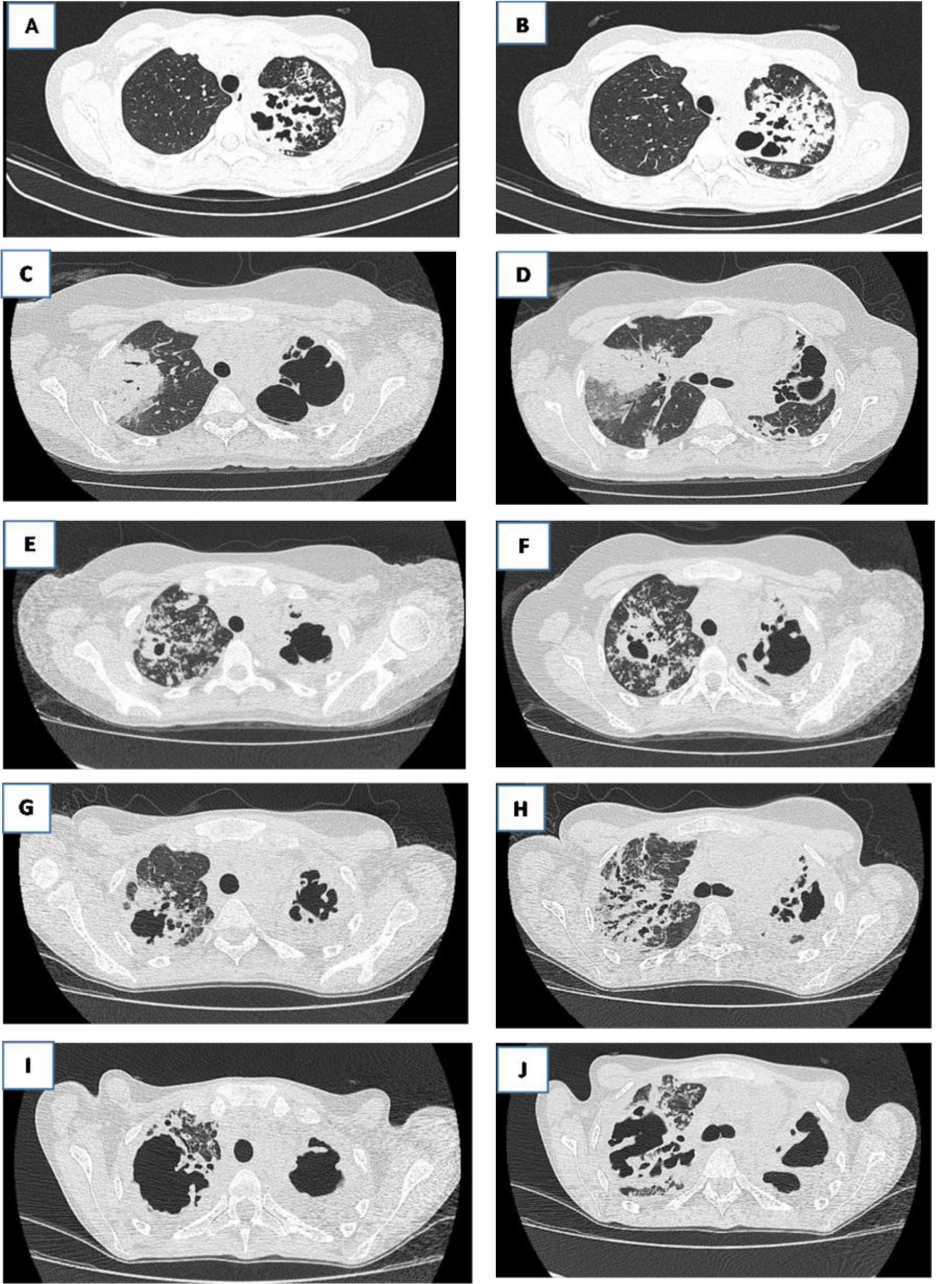
The evolution of chest CT scan images during treatment shows extensive pulmonary involvement: A and B) January 2017; C and D) September 2018; E and F) October 2019; G and H) June 2021; I and J) January 2022.

A 26-year-old healthy female was referred from primary healthcare to the outpatient clinic of Maria Aparecida Pedrossian University Hospital, Campo Grande municipality, Mato Grosso do Sul State, Brazil, which is a secondary referral center for TB. She had no history of smoking, alcoholism, or illicit drug use. The initial chest computed tomography (CT) revealed cavitation and intense inflammation (Figures 1A and 1B). She began treatment for pulmonary tuberculosis (PTB) in January 2017, as confirmed by sputum cultures of *Mycobacterium tuberculosis* (Mtb), which were sensitive to all drugs (Table 1). She received rifampicin, isoniazid, pyrazinamide, and ethambutol (RIPE) for two months and rifampicin and isoniazid (RI) for only two months. She then discontinued treatment because of clinical improvement.

**Table 1:** Characterization of the clinical and laboratory follow-up stages of the case.

Treatment cycle	Start date	End date	Sputum AFB test – date: result	MTb Culture – date: result – methodology	Antibiotic sensitivity test – result (methodology)	Result of RMT* – date: result; sensitivity to R	Treatment administered	Criteria for treatment suspension
First cycle	January 2017	May 2017	01/10/2017: negative	01/10/2017 positive – MGIT	Sensitive to SM and RIE (MGIT)	Not performed	RIPE for 2 months and RI for 2 months	The patient decided to discontinue treatment in the fourth month, reporting clinical improvement
Second cycle	October 2017	April 2018	10/09/2017: positive (+/4+)	10/09/2017: positive – Ogawa-Kudoh	Not performed	Not performed	RIPE for 2 months and RI for 4 months	Resolution of symptoms, with weight gain (10 kg) and improvement in laboratory tests. Did not collect sputum for treatment monitoring due to absence of secretion
Third cycle	September 2018	July 2019	09/05/2019: (+/4+)	09/05/2019: positive – MGIT 05/25/2019: negative – Ogawa-Kudoh	Sensitive to RIE and resistant to SM (MGIT).	Not performed	RIPE for 2 months and RI for 7 months	The patient showed significant clinical improvement but did not provide sputum for treatment monitoring due to absence of secretion
Fourth cycle	October 2019	Extension of the induction phase by four months and loss to follow-up due to the pandemic.	09/19/2019: positive (+/4+)	09/19/2019: positive – MGIT 08/10/2019: negative – Ogawa-Kudoh	Sensitive to RIE and resistant to SM (MGIT)	Not performed	RIPE for 4 months and RI for an unaccounted period due to loss to follow-up	In March 2020, she had satisfactory clinical progress when she confirmed pregnancy. Subsequently, she was lost to follow-up due to the suspension of all outpatient services during the pandemic
Fifth cycle	February 2021 (return to outpatient clinic in person).	Around December 2021 to February 2022.	02/06/2021: positive (++/4+) 06/17/2021: negative 08/16/2021: positive (+/4+)	02/06/2021: negative – Ogawa-Kudoh) 06/17/2021: negative – MGIT 08/16/2021: positive – MGIT	Sensitive to RIE and resistant to SM (MGIT)	06/17/2021: MTb detectable; Sensitive to R 08/16/2021:Mtb detectable; Sensitive to R	RIPE for 10 to 12 months	There was progressive clinical improvement until the last quarter of 2021, maintained on a four-drug regimen. Currently, there has been a delay in the patient’s outpatient follow-up
Sixth cycle	February 2022	Did not complete treatment. Was maintained under home care service.	01/27/2022: positive (+/4+)	01/27/2022: positive – MGIT	Sensitive to RIE and resistant to SM; MGIT Sensitive to RI; LPA *Detection of Mtb by partial sequencing of 16S rRNA and hsp65 genes. Partial sequencing of rpoB gene did not detect genetic alterations. Method: Direct sequencing using the Sanger method. Acquisition platform: ABI3500 Genetic Analyzer (Applied Biosystems)	03/29/2022: MTb detectable, sensitive to R	Bedaquiline, linezolid, terizidone, and levofloxacin for 6 months, followed by continuation with linezolid, terizidone, and levofloxacin for another 12 months	The patient was hospitalized for DOT and respiratory rehabilitation, but also experienced clinical failure with persistent acid-fast bacilli (AFB) in sputum at the sixth month of treatment
Treatment cycle with intravenous drugs	August 2022	Discharge in August 2022 upon request after seven days of hospitalization	08/21/2022: positive (+/4+)	08/21/2022: positive – MGIT 08/10/2022: positive – MGIT	08/21/2022: sensitive to RIE and resistant to SM (MGIT) 08/10/2022: sensitive to RIE and resistant to SM (MGIT)	08/21/2022: MTb detectable; Sensitive to R	Amikacin, levofloxacin, linezolid, amoxicillin-clavulanate, and meropenem	-

MGIT = mycobacterial growth indicator tube; MTB = *Mycobacterium tuberculosis*; TB = tuberculosis; RIE = rifampicin, isoniazid, ethambutol; RIPE = rifampicin, isoniazid, pyrazinamide, ethambutol; SM = streptomycin; PCR = Polymerase chain reaction; AFB = acid-fast bacilli; DOT = Directly observed therapy; *Molecular biology, RMT = rapid molecular testing, Real-time PCR (Xpert MTB/RIF kit [Cepheid]).

The patient reported working as a manicurist, living in an urban area with her three young children, and had partially completed her high school education. She also received family support and had easy access to health services. All her contacts were referred to a primary healthcare unit for follow-up. Treatment for TB was provided to one of her children, whereas the others received prophylaxis treatment.

In May 2017, she returned with consumptive syndrome, fever, and respiratory symptoms and tested positive for AFB and Mtb in sputum (Table 1) with negative HIV serology. The patient visited the TB reference outpatient clinic at the Maria Aparecida Pedrossian University Hospital and was welcomed by the healthcare team. She underwent a total of six months treatment: two months of RIPE and four months of RI, which led to symptom resolution and a 10 kg weight gain. She was categorized as having an intermediate risk of treatment abandonment due to her age and previous TB treatment abandonment^4^.

In September 2018, her PTB symptoms recurred with positive sputum AFB results (Table 1) and worsening cavitation on chest CT (Figures 1C and 1D). RIPE treatment was resumed, and the patient’s condition improved clinically. By May 2019, despite the clinical improvement, the patient remained AFB-positive, and chest computed tomography (CT) revealed active disease. The RI continuation phase was extended to nine months, ending in July 2019. The presence of multidrug-resistant tuberculosis was ruled out by identifying RIE-sensitive Mtb in a sputum culture (Table 1).

In October 2019, the patient returned with respiratory complaints, weight loss, and AFB-positive sputum (Table 1). RIPE was reintroduced, and chest CT revealed an active inflammatory process (Figures 1E and 1F). The CD4+ T cell count (382 cells/UL) and autoimmune tests showed no other underlying diseases. After two months of treatment, the patient remained AFB-positive, prompting an extended intensive phase before switching to RI (Table 1).

Associated conditions such as chronic pulmonary aspergillosis, histoplasmosis, paracoccidioidomycosis, and nontuberculous mycobacterial disease were extensively pursued and ruled out.

In March 2020, the patient showed significant clinical improvement when she was confirmed pregnant and started prenatal care. The patient had hyperemesis gravidarum and was hospitalized in May 2020, remaining positive for AFB (Table 1). The COVID-19 pandemic disrupted her outpatient follow-up, although communication continued via mobile apps (e.g., WhatsApp). As the pandemic worsened, her follow-up was completely suspended. In October 2020, she delivered a healthy full-term newborn.

By February 2021, she returned emaciated and AFB-positive. Despite restarting RIPE, her condition worsened, and by June 2021, she remained Mtb-positive with active disease on chest CT (Figure 1G and 1H). We maintained the RIPE regimen throughout the treatment period and continued investigations to determine the reasons for drug non-absorption despite finding persistent antibiotic sensitivity in the cultures.

In January 2022, significant progression of the lung lesions (Figures 1I and 1J) required home-based oxygen therapy. After considerable clinical failure, a new regimen of bedaquiline, linezolid, terizidone, and levofloxacin was initiated in February 2022.

During this period, the Central Laboratory of Mato Grosso do Sul (LACEN-MS) sent the strain for molecular biology testing to the Central Laboratory of Public Health of Distrito Federal (LACEN-DF). Mtb was detected by partial sequencing of the 16S rRNA and hsp65 genes. Partial sequencing analysis of the rpoB gene did not detect genetic alterations capable of justifying the bacterial resistance phenotype in the analyzed sample. In conclusion, these results were consistent with those of an earlier Line Probe Assay (LPA), which showed sensitivity to RI. Direct sequencing following the Sanger method was performed using the ABI3500 Genetic Analyzer (Applied Biosystems, Waltham, MA, USA).

She was hospitalized for respiratory failure and underwent tentative DOT for three months at a rehabilitation hospital. After discharge, which was requested by the patient and her family, her condition worsened, and she remained AFB-positive, indicating treatment failure. At this stage, the team attempted another hospitalization for the administration of intravenous medications (amikacin, levofloxacin, linezolid, amoxicillin-clavulanate, and meropenem). However, the patient was resistant to hospitalization and stayed for only seven days before requesting discharge.

In this context, the following facts were reported to the healthcare team during hospitalization for intravenous medication administration: 1) although the patient initially received oral medication in all the treatment cycles, and she showed clinical improvement, she discontinued its use because she felt unwell when taking the pills and believed that the medications would worsen her symptoms, and 2) a family member reported that illicit substance use was also part of the home environment. The information about lack of adherence to medications in all treatment cycles and illicit substance use were never reported during consultations and was only disclosed to the team during the attempt at intravenous treatment. After the team became aware of illicit substance use, the patient was reclassified as having a high risk of treatment abandonment.

Expressing their desire to be discharged, the families acknowledged the severity of their condition. Terminal care was provided for the extensive disease activity and cavitation. The patient was discharged for home follow-up and died in October 2022.

## DISCUSSION

This study describes a case of PTB in a young patient who experienced frequent recurrence and worsening of pulmonary lesions after completing several treatment cycles. The key characteristics are as follows:

1. Voluntary return to a specialized TB clinic after initial treatment dropout in 2017;
2. Intermediate risk for treatment abandonment due to age and TB retreatment;
3. Regular attendance at appointments and communication with care teams via social media during the pandemic.
4. No reported intolerance, irregular use, or adverse events related to medications.
5. No history of drug use or depression.
6. Consistent adherence and understanding of prescribed medications and investigations for treatment failure causes.

Overall, in the initial phase, the social and economic contexts did not appear significant for non-adherence to medication, as the patient was actively working and had good family support. Unfortunately, the described social context, including the patient’s difficulty understanding the importance of adherence and the use of illicit substances, directly contributed to the reported outcome. What caught our team’s attention was the difficulty of achieving effective communication with the primary healthcare unit, as effective coordination could be a crucial tool for building a successful STP.

Considering that TB shows a good prognosis and evolution to cure in most cases when treatment is performed appropriately, and Mtb is sensitive to the drugs used, the reported clinical evolution has become a challenge for the entire team^6^.

When she was admitted to our hospital, we registered her first treatment as abandoned because of the suspension of treatment for more than 30 days^7^. We highlight the possibility of treatment for four months in HIV-uninfected patients with evidence of TB infection, negative sputum cultures, and symptomatic and/or radiographic improvements in the absence of an alternative diagnosis. However, this was not possible in our report^8^.

A study conducted by our team from 2012 to 2019 on the risk factors associated with TB treatment abandonment in Mato Grosso do Sul State provided important data for understanding this case. In our outpatient clinic, smoking status, disease retreatment, and high-risk scores were associated with treatment abandonment. Of the patients who abandoned treatment, 87.5% had a high- or intermediate-risk score, and 12.5% had a low-risk score^4^.

Although the patient was classified as having an intermediate risk based on the treatment abandonment score, being younger than 30 years, and previous treatment for TB, in general, during the treatments performed by the care team, the possibility of abandonment at any time was not raised, as we considered the patient to be adherent and always responsive to questions about the possibility of failure of the scheme. She was often accompanied by a family member who confirmed the correct use of medications. After learning about the use of illicit substances, the patient was reclassified as having a high-risk score.

In the initial treatment cycles, the patient showed significant clinical improvement but immediate access to the test results was not always available. The patient had difficulty providing sputum samples, and a positive result was often accompanied by marked clinical improvement. Culture results were also intermittently accessible and, when available, showed susceptibility to all drugs. The team’s confidence in the patient’s regular medication adherence, reinforced by the presence of a family member at her regular appointments and her persistent denial of any difficulties or adverse events related to medication use, led the team to investigate other causes of persistently positive AFB test results without considering clinical failure.

As the patient presented with progressive cavitations in tomographic images and persistence of positive sputum smear microscopy results, these were considered important factors that could hinder the treatment response. The interior of the cavities is poorly vascularized due to vascular necrosis, which leads to poor penetration of antimycobacterial medications^9^. Suboptimal drug levels can also lead to the selection of drug-resistant mutants^10^, which was not observed in the present study.

An important factor that hindered the patient’s follow-up was the beginning of the COVID-19 pandemic. Despite the team’s initiative to maintain contact for remote follow-up, we could not assess clinical worsening in person. The pandemic has contributed to a decrease in the number of registered TB cases compared to the data observed in 2019, with a decrease in the diagnosis and treatment of the disease worldwide being the leading cause^11^.

During the pandemic, the patient became pregnant, and even during the normal gestation period, there was a relative suppression of Th1-type cytokines in the lymphocyte response, leading to a prevalence of the Th2-type response, called gestational immunosuppression^12^. In addition, hospitalization due to hyperemesis gravidarum has been reported, which is another factor that may have contributed to the lack of treatment adherence.

Immunosuppressive conditions were also investigated; however, the results were not consistent with those of specific rheumatological diseases. A CD4+ T cell count of 382 cells/UL raised the possibility of idiopathic low CD4+ T cell syndrome, defined as persistent CD4+ T cell lymphopenia in the absence of HIV-1 infection or any other cause of immunodeficiency. CD4+ T cell counts are usually below 300 cells/µL or less than 20% of total lymphocytes on more than one occasion, usually two or three months apart. Another possibility is a reduced CD4+ T cell count in a transient manner due to prolonged infection, which results in the consumption of these cells^13^. The patient probably showed a transient reduction in CD4+ T cells levels due to persistent TB activity.

Sputum cultures always identified Mtb as sensitive to all drugs used for treatment. During the investigation, the strain was sent by LACEN-MS to LACEN-DF for sequencing, which did not reveal any genetic alterations in the samples that could explain its clinical resistance. This investigation was conducted to rule out the possibility of infection by a strain with a resistant genotype and sensitive phenotype. Beijing-type Mtb strains have a greater capacity of resisting TB treatment even in the absence of drug resistance. This strain is highly virulent and is associated with treatment failure^14^. In Brazil, the Beijing Mtb strain was first identified in 2002 and continues to cause only 0.8% of TB cases^15^.

DOT is a valuable tool for linking patients to health services, ensuring observation of medication intake and reinforcing adherence^4^. From the beginning of follow-up with our team, the treatment was self-administered and considered regular because the patient attended consultations and appeared to have a good understanding of the need for treatment adherence. Although the patient did not undergo DOT, follow-up was performed by a team in the basic health unit (BHU). However, there was no regular communication between the BHU and our team, which hindered perceiving irregularities. Adherence to consultations and systematic family follow-ups are not substitutes for treatment adherence, as observed in our case report.

In complex cases, such as this, a STP is indicated, which is an important strategy for organizing care focused on the individual or family, considering the patient’s particularities. The STP is built between the multidisciplinary health team and the patient, with responsibilities assigned, seeking to value the individual’s life history and bring them to the center of care. It is an important tool for detecting the main factors that contribute to low adherence to and abandonment of TB treatment^6^. According to World Health Organization (WHO), one of the pillars of ending epidemics by 2030 is prevention and integrated and patient-centered care^7,16^.

Despite all efforts by the care team to understand the reason for the refractoriness of the treatment, the clinical evolution was unfavorable, and we believe that poor adherence to treatment was the main cause.

This case provides an excellent learning experience for everyone and demonstrates the importance of comprehensively assessing a patient’s needs, including the participation of family members. It is essential to view the patient as a whole, and not just focus on the disease, regardless of its severity and complexity. Our case report demonstrates that each patient is unique, with particularities related to their life history, beliefs, and stigmas related to the disease, family structure, and emotional state.

It is extremely important to offer psychological support to all sick patients, especially those facing serious, complex, and stigmatizing illnesses and prolonged treatment. Emotional support is essential for patients and their family members to understand the disease, treatment, and responsibilities as fundamental parts of the process^6^. These approach steps, as discussed above, failed to detect vulnerabilities and psychological disorders related to the patient and her family during the last hospitalization. We believe that a systematic social and psychological approach, as well as greater integration with the BHU, could have aided detect these factors and contributed to a different outcome.

**Figure 2:**
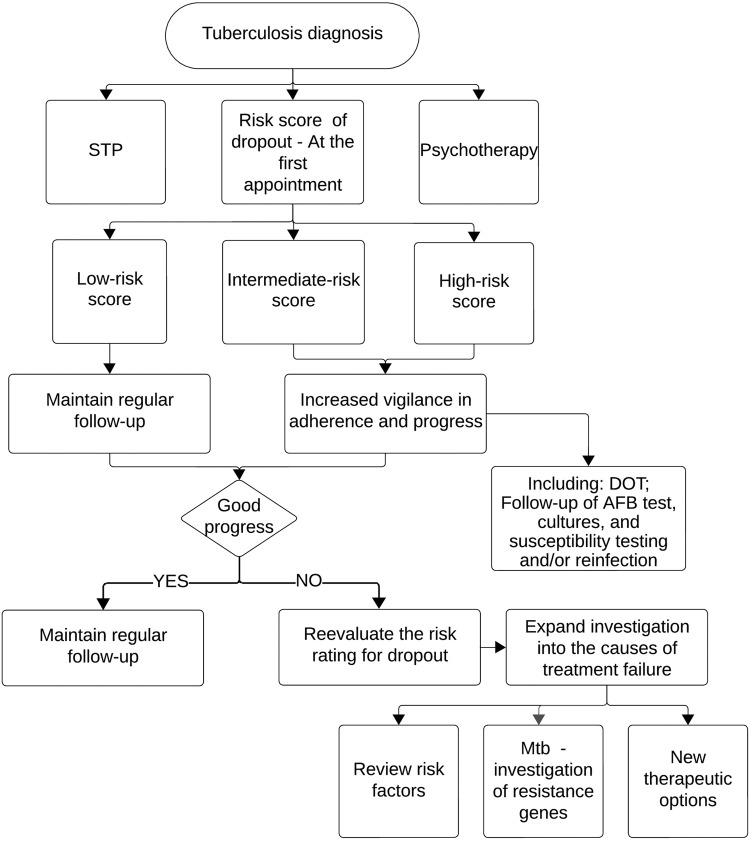
Flowchart for the approach to tuberculosis cases. STP = Singular therapeutic project; DOT = Direct observed therapy; AFB = Acid-fast bacillus; Mtb = *Mycobacterium tuberculosis*.

This study led us to propose strategies (Figure 2) to address complex cases.

## CONCLUSION

An important tool is to apply the risk score for TB treatment abandonment to every patient upon admission. STP should include DOT and both clinical and laboratory monitoring, with sputum smear and culture control, as well as systematic coordination with the reference BHU for active monitoring of patients with high or intermediate scores. Associated with this, they offer psychological follow-ups to patients and their families, which can aid provide emotional support, identify weaknesses, and establish alternatives to cope with the disease and its adversities, thus improving treatment adherence.
